# Supplementary material for: Influence of pain on the quality of life in patients with venous ulcers: Cross-sectional association and correlation study in a brazilian primary health care lesions treatment center
Source: PLoS One. 2023 Aug 15;18(8):e0290180. doi: 10.1371/journal.pone.0290180 (PMC10426926; doi:10.1371/journal.pone.0290180)
Supplement: S1 Table — a Spearman’s Rho test; b p-value for Spearman’s coefficient; c p-value not shown in test due to maximum correlation strength; Correlation levels: r ≤ 0.29 (weak); 0.29 > r ≤ 0.49 (moderate); r ≥ 0.50 (strong). (DOCX) [file pone.0290180.s001.docx]

**S1 Table.** Correlation between QoL variables (SF-36) among participants according to pain levels (VAPS)

| **Pain Levels (VAPS)** | **QoL (SF-36)** | **QoL (n= 103)** | | | | | | | | | | |
| --- | --- | --- | --- | --- | --- | --- | --- | --- | --- | --- | --- | --- |
|  |  | Physical functioning | Physical role functioning | Pain | General health perceptions | Vitality | Social role functioning | Emotional role functioning | Mental Health | Physical health Dimension | Mental Health Dimension | Total Score |
|  |  | *r*^a^ (p)^b^ | *r* (p) | *r* (p) | *r* (p) | *r* (p) | *r* (p) | *r* (p) | *r* (p) | *r* (p) | *r* (p) | *r* (p) |
| Absent/Mild (n= 46) | Physical functioning | - | 0.63 (<0.001) | -0.58 (<0.001) | -0.09 (0.955) | 0.09 (0.556) | 0.19 (0.192) | 0.61 (<0.001) | 0.51 (<0.001) | 0.54 (<0.001) | 0.52 (<0.001) | 0.64 (<0.001) |
|  | Physical role functioning | 0.63 (<0.001) | - | -0.58 (<0.001) | 0.22 (0.140) | 0.01 (0.942) | 0.11 (0.457) | 0.93 (<0.001) | 0.35 (0.016) | 0.66 (<0.001) | 0.61 (<0.001) | 0.66 (<0.001) |
|  | Pain | -0.58 (<0.001) | -0.58 (<0.001) | - | 0.26 (0.083) | -0.22 (0.141) | 0.003 (0.986) | -0.58 (<0.001) | -0.51 (<0.001) | -0.1 (0.552) | -0.33 (0.026) | -0.21 (0.152) |
|  | General health perceptions | -0.01 (0.955) | 0.22 (0.140) | 0.25 (0.83) | - | -0.22 (0.130) | -0.23 (0.044) | 0.19 (0.208) | -0.08 (0.584) | 0.63 (<0.001) | 0.37 (0.011) | 0.43 (0.003) |
|  | Vitality | 0.09 (0.556) | 0.01 (0.942) | -0.22 (0.141) | -0.22 (0.130) | - | 0.24 (0.110) | 0.03 (0.808) | 0.34 (0.019) | 0.17 (0.249) | 0.35 (0.016) | 0.27 (0.066) |
|  | Social role functioning | 0.19 (0.192) | 0.11 (0.457) | 0.002 (0.986) | -0.23 (0.044) | 0.24 (0.110) | - | 0.12 (0.432) | 0.19 (0.208) | 0.14 (0.332) | 0.43 (0.003) | 0.41 (0.004) |
|  | Emotional role functioning | 0.61 (<0.001) | 0.93 (<0.001) | -0.58 (<0.001) | 0.19 (0.208) | 0.03 (0.808) | 0.12 (0.432) | - | 0.37 (0.010) | 0.62 (<0.001) | 0.62 (<0.001) | 0.62 (<0.001) |
|  | Mental Health | 0.51 (<0.001) | 0.35 (0.016) | -0.50 (<0.001) | -0.08 (0.584) | 0.34 (0.019) | 0.19 (0.208) | 0.37 (0.010) | - | 0.22 (0.130) | 0.63 (<0.001) | 0.47 (0.001) |
|  | Physical health Dimension | 0.54 (<0.001) | 0.66 (<0.001) | -0.09 (0.552) | 0.63 (<0.001) | 0.17 (0.249) | 0.14 (0.332) | 0.62 (<0.001) | 0.22 (0.130) | - | 0.76 (<0.001) | 0.91 (<0.001) |
|  | Mental Health Dimension | 0.52 (<0.001) | 0.61 (<0.001) | -0.33 (0.026) | 0.37 (0.011) | 0.35 (0.016) | 0.43 (0.003) | 0.62 (<0.001) | 0.63 (<0.001) | 0.76 (<0.001) | - | 0.92 (<0.001) |
|  | Total Score | 0.64 (<0.001) | 0.66 (<0.001) | -0.21 (0.152) | 0.43 (0.003) | 0.27 (0.066) | 0.41 (0.004) | 0.62 (<0.001) | 0.47 (0.001) | 0.91 (<0.001) | 0.92 (<0.001) | - |
| Moderate (n= 9) | Physical functioning | - | 0.78 (0.012) | -0.84 (0.004) | 0.02 (0.957) | -0.33 (0.385) | 0.64 (0.065) | 0.78 (0.012) | 0.60 (0.085) | 0.78 (0.012) | 0.81 (0.009) | 0.84 (0.005) |
|  | Physical role functioning | 0.78 (0.012) | - | -0.79 (0.010) | 0.17 (0.653) | -0.54 (0.129) | 0.27 (0.476) | 1.000 (-) ^c^ | 0.36 (0.336) | 0.87 (0.002) | 0.86 (0.003) | 0.86 (0.003) |
|  | Pain | -0.84 (0.004) | -0.79 (0.010) | - | 0.14 (0.716) | 0.19 (0.612) | -0.37 (0.317) | -0.79 (0.010) | -0.46 (0.208) | -0.71 (0.032) | -0.80 (0.010) | -0.70 (0.033) |
|  | General health perceptions | 0.02 (0.957) | 0.17 (0.653) | 0.14 (0.716) | - | -0.43 (0.240) | 0.080 (0.838) | 0.17 (0.653) | -0.11 (0.778) | 0.53 (0.141) | 0.45 (0.220) | 0.45 (0.220) |
|  | Vitality | -0.33 (0.385) | -0.54 (0.129) | 0.19 (0.612) | -0.43 (0.240) | - | -0.39 (0.303) | -0.54 (0.129) | 0.04 (0.916) | -0.62 (0.073) | -0.45 (0.219) | -0.66 (0.051) |
|  | Social role functioning | 0.64 (0.065) | 0.27 (0.476) | -0.37 (0.317) | 0.08 (0.838) | -0.39 (0.303) | - | 0.27 (0.476) | 0.14 (0.723) | 0.45 (0.224) | 0.37 (0.329) | 0.53 (0.145) |
|  | Emotional role functioning | 0.78 (0.012) | 1.000 (-) ^c^ | -0.79 (0.010) | 0.17 (0.653) | -0.54 (0.129) | 0.27 (0.476) | - | 0.36 (0.363) | 0.87 (0.002) | 0.86 (0.003) | 0.86 (0.003) |
|  | Mental Health | 0.60 (0.085) | 0.36 (0.336) | -0.46 (0.208) | -0.11 (0.778) | 0.041 (0.916) | 0.14 (0.723) | 0.36 (0.336) | - | 0.38 (0.310) | 0.44 (0.239) | 0.38 (0.307) |
|  | Physical health Dimension | 0.78 (0.012) | 0.87 (0.002) | -0.71 (0.032) | 0.53 (0.141) | -0.62 (0.073) | 0.45 (0.224) | 0.87 (0.002) | 0.38 (0.310) | - | 0.96 (<0.001) | 0.98 (<0.001) |
|  | Mental Health Dimension | 0.81 (0.009) | 0.86 (0.003) | -0.80 (0.010) | 0.45 (0.220) | -0.45 (0.219) | 0.37 (0.329) | 0.86 (0.003) | 0.44 (0.239) | 0.96 (<0.001) | - | 0.92 (0.001) |
|  | Total Score | 0.84 (0.005) | 0.86 (0.003) | -0.71 (0.033) | 0.45 (0.220) | -0.66 (0.051) | 0.52 (0.145) | 0.86 (0.003) | 0.38 (0.307) | 0.98 (<0.001) | 0.92 (0.001) | - |
| Severe (n= 48) | Physical functioning | - | 0.70 (<0.001) | -0.75 (<0.001) | -0.39 (0.006) | 0.03 (0.812) | -0.07 (0.648) | 0.61 (<0.001) | 0.43 (0.002) | 0.72 (<0.001) | 0.51 (<0.001) | 0.74 (<0.001) |
|  | Physical role functioning | 0.70 (<0.001) | - | -0.70 (<0.001) | -0.36 (0.013) | -0.02 (0.859) | -0.18 (0.214) | 0.91 (<0.001) | 0.40 (0.004) | 0.82 (<0.001) | 0.76 (<0.001) | 0.85 (<0.001) |
|  | Pain | -0.75 (<0.001) | -0.70 (<0.001) | - | 0.53 (<0.001) | -0.19 (0.184) | -0.06 (0.655) | -0.68 (<0.001) | -0.63 (<0.001) | -0.41 (0.003) | -0.63 (<0.001) | -0.54 (<0.001) |
|  | General health perceptions | -0.39 (0.006) | -0.36 (0.013) | 0.53 (<0.001) | - | 0.10 (0.507) | -0.04 (0.753) | -0.33 (0.022) | -0.13 (0.367) | 0.03 (0.839) | 0.013 (0.931) | -0.06 (0.672) |
|  | Vitality | 0.03 (0.812) | -0.02 (0.859) | -0.19 (0.184) | 0.10 (0.507) | - | 0.09 (0.555) | -0.01 (0.951) | 0.57 (<0.001) | -0.005 (0.970) | 0.33 (0.019) | 0.11 (0.465) |
|  | Social role functioning | -0.07 (0.648) | -0.18 (0.214) | -0.06 (0.655) | -0.04 (0.753) | 0.09 (0.555) | - | -0.22 (0.131) | 0.05 (0.730) | -0.22 (0.127) | 0.037 (0.805) | -0.10 (0.485) |
|  | Emotional role functioning | 0.61 (<0.001) | 0.91 (<0.001) | -0.68 (<0.001) | 0.33 (0.022) | -0.01 (0.951) | -0.22 (0.131) | - | 0.38 (0.008) | 0.73 (<0.001) | 0.83 (<0.001) | 0.83 (<0.001) |
|  | Mental Health | 0.43 (0.002) | 0.40 (0.004) | -0.63 (<0.001) | -0.13 (0.367) | 0.57 (<0.001) | 0.05 (0.730) | 0.38 (0.008) | - | 0.26 (0.072) | 0.64 (<0.001) | 0.43 (0.002) |
|  | Physical health Dimension | 0.72 (<0.001) | 0.82 (<0.001) | -0.41 (0.003) | 0.03 (0.839) | -0.005 (0.970) | -0.22 (0.127) | 0.73 (<0.001) | 0.26 (0.072) | - | 0.69 (<0.001) | 0.94 (<0.001) |
|  | Mental Health Dimension | 0.51 (<0.001) | 0.76 (<0.001) | -0.63 (<0.001) | 0.01 (0.931) | 0.33 (0.019) | 0.03 (0.805) | 0.83 (<0.001) | 0.64 (<0.001) | 0.69 (<0.001) | - | 0.83 (<0.001) |
|  | Total Score | 0.74 (<0.001) | 0.85 (<0001) | -0.54 (<0.001) | -0.06 (0.672) | 0.11 (0.465) | -0.10 (0.485) | 0.83 (<0.001) | 0.43 (0.002) | 0.94 (<0.001) | 0.83 (<0.001) | - |

a Spearman's Rho test

b p-value for Spearman's coefficient.

c p-value not shown in test due to maximum correlation strength.

Correlation levels: r ≤ 0.29 (weak); 0.29 > r ≤ 0.49 (moderate); r ≥ 0.50 (strong);
