# Supplementary material for: Influence of pain on the quality of life in patients with venous ulcers: Cross-sectional association and correlation study in a brazilian primary health care lesions treatment center
Source: PLoS One. 2023 Aug 15;18(8):e0290180. doi: 10.1371/journal.pone.0290180 (PMC10426926; doi:10.1371/journal.pone.0290180)
Supplement: Attachment — Submitted filename: Response to Reviewers.docx [file pone.0290180.s002.docx]

**Response to Reviewers**

**Reviewer #1:**

Comments to the Autor:

This is an interesting paper and will be of interest to those working in the área of chronic wounds and quality of life (QoL). There a few points of clarification that would help the reader:

Materials and Methods

- Ethical aspects:

Please, explain in “Before the application of any instrument, the objective of the study was explained and a signature was requested in the Informed Consent Form” Did the patients signe the Informed Consent form?

**Answer:** The term was signed in writing by all participants. To make this clearer, we have added this information to the end of the description of the ethical aspects.

- Instruments and variables:

Please, explain why did you use SF-36 and not a venous ulcer quality of life questionaire?

**Answer:** We prefer to choose the SF-36 instead of other instruments because we consider it more complete due to the number of aspects evaluated. We add this information to the methodology in the body of the text.

Did the patients take any analgesic?

**Answer:** Before the survey, participants were asked if they had used any type of analgesic on the day of the interview. If they answered in the affirmative, the collection would be scheduled for another time. However, none of the individuals reported that they used it. We add this information in the body of the text in the topic "Data collection and availability". Thank you.

- Data collection and availability

Please, explain how much time does it normally take from the contact with the patient to the interview?

**Answer:** Medical follow-up appointments and dressing changes occurred weekly with each of the participants, according to the service's agenda. The researchers attended the days of the consultations without previously notifying the participants and invited them to participate in the research. This information was added to the text of the manuscript to make it clearer.

Results

Please, In Tables 2 and 3, check the numbers are located in their rows.

**Answer:** Some numbers were misaligned with the tables. We made the necessary adjustments. Thank you.

**Reviewer #2:**

A temática é atual, pertinente e interessante, pois, atualmente, observa-se um crescimento no diagnóstico das úlceras venosas (UV) e das condições que podem causá-las. O assunto já é bem explorado em estudos internacionais e já existem alguns estudos nacionais abordando a temática. Sugere-se que os autores destaquem os achados inéditos ou pouco explorados.

A leitura do manuscrito é interessante, entretanto, destaco os seguintes pontos:

- Sugiro revisão no título do manuscrito, pois trata-se de um estudo pontual, realizado em um único centro, questiona-se se refletiria a realidade de toda a atenção primária brasileira, como consta no título.

**Answer:** Realizamos a alteração sugerida delimitando o cenário para um centro de tratamento de lesões.

- O resumo apresenta os principais achados do manuscrito, entretanto, faço a mesma observação em relação ao título, questiona-se se um único estudo pontual poderia refletir a realidade brasileira?

**Answer:** Fizemos uma alteração na redação direcionando o objetivo para a realização do estudo no centro de tratamento de lesões.

- A introdução é objetiva, interessante e chama a atenção para o problema, trazendo dados sobre a epidemiologia as UV. A justificativa para a realização do estudo está presente. O objetivo do estudo é descrito, porém, sugiro revisão em relação ao alcance do estudo (realidade brasileira?).

**Answer:** Fizemos os ajustes a exemplo das observações anteriores.

-O método escolhido é adequado para se responder ao objetivo do estudo. A seção "Métodos" foi escrita de forma bem detalhada. Sugiro a utilização da instrução STROBE, como um guia para a elaboração do artigo.

**Answer:** O recurso do checklist STROBE foi utilizado anteriormente e enviado preenchido junto à submissão inicial. No entanto, fizemos uma nova revisão diante de vosso comentário.

-Há informações repetidas sobre a população do estudo.

**Answer:** Identificamos algumas informações que realmente estavam repetidas. Fizemos os ajustes necessários.

- Menciona-se a utilização de um "instrumento" para a coleta de dados sociodemográficos, porém, questiona-se se houve um processo formal de validação desse instrumento. Caso não seja validado, sugiro que seja utilizado o termo roteiro de coleta de dados.

**Answer:** Como se trata de um texto escrito em inglês, substituímos pelo termo “data collection script”.

- Sugiro a inserção da referência adotada para a classificação das forças de correlação.

**Answer:** A mesma referência que utilizamos para os demais parâmetros estatísticos foi a que escolhemos para a as forças de correlação. Para ficar mais claro, acrescentamos a mesma citação logo após a descrição dos parâmetros da força de correlação.

- Questiona-se se o uso de medicamentos psicoativos foi investigado, pois seu uso poderia comprometer a investigação de constructos subjetivos, como a QV. A mesma questão pode estar relacionada ao diagnóstico de depressão.

**Answer:** De fato, o uso de psicoativos não foi investigado. Concordamos com a possibilidade de comprometer as interpretações. Fizemos um acréscimo sobre essa reflexão nas limitações do trabalho. No entanto, os participantes foram perguntados sobre o uso de medicamentos analgésicos, uma vez que o estudo se dedica a investigar os aspectos da dor.

- Na seção "Resultados", os autores repetem no corpo do texto, por várias vezes, os resultados apresentados nas tabelas, o que poderia tornar a leitura pouco fluida. Os resultados devem ser apresentados na tabela ou no texto, não em ambos.

**Answer:** Retiramos todos os valores numéricos do texto que estão presentes na tabela.

- Na tabela 1, os autores mencionam o status mental, bem como o uso de medicações, porém não fica claro seu significado ou quais medicações utilizadas foram investigadas. Sugiro que essas informações sejam detalhadas no texto subsequente à tabela.

**Answer:** Essa variável se refere a medicações de uso diário em geral. O participante foi perguntado simplesmente se usa algum medicamento diariamente, para tratamento de alguma doença crônica, com hipertensão ou Diabetes. Acrescentamos uma legenda depois da tabela 1.

- A formatação das tabelas necessita de revisão. Questiona-se se não seria mais adequada a apresentação das medianas nas tabelas ao invés das médias, pois foram utilizados testes não paramétricos. Além disso, os testes utilizados não permitem, por exemplo, verificar as diferenças inter/intragrupos entre os domínios do SF-36 e os participantes com dor ausente, moderada e forte. A utilização de uma análise estatística mais robusta poderia contribuir com o avanço do conhecimento e visibilidade do artigo.

**Answer:** Fizemos os ajustes sugeridos para a formatação da Tabela 3. Nesse caso, não só mostramos a mediana representada pelo percentil 50, mas também a distribuição entre os três percentis, a fim de se padronizar o desenho com o das tabelas anteriores. Realizamos também uma análise adicional a fim de contemplar o apontamento da comparação inter/intragrupos. Foram acrescentadas as Tabelas 5, 6 e 7 que foram montadas individualmente (cada uma com um nível de intensidade da dor) e ranqueadas de acordo com a força de correlação e nível de significância entre as variáveis da qualidade de vida. A fim de melhorar a compreensão dos resultados, suprimimos algumas variáveis. No entanto, adicionamos como arquivo de suporte a Tabela S1 com todos os dados, sem supressão. Acreditamos que com essa análise, além de deixar nossos resultados mais robustos e esclarecedores, contemplamos integralmente vosso apontamento.

- Na seção "Discussão", os autores trazem vários artigos, atuais e pertinentes, para fundamentar sua discussão, explorando as associações encontradas no estudo com a literatura disponível sobre o assunto, o que tornou a discussão ampla e interessante. Entretanto, questiona-se se realmente algumas das afirmações realizadas pelos autores são suportadas pelos resultados obtidos. Sugiro revisão.

**Answer:** Fizemos uma nova revisão e ajustamos a escrita de alguns fragmentos que poderiam ser interpretados equivocadamente como uma inferência de causa e efeito. Se ainda assim, perceber que o problema continua, peço gentilmente que aponte mais especificamente onde estão as afirmações que se encontram possivelmente sem a devida sustentação.

- As limitações são apresentadas ao final do estudo. A conclusão responde ao objetivo do estudo. Os autores apresentam 37 referências, sendo 25 dos últimos cinco anos.

**Answer:** Fizemos algumas alterações mediante solicitação dos revisores, assim como do editor.
